# Exploring the role of hub and network dysfunction in brain connectomes of schizophrenia using functional magnetic resonance imaging

**DOI:** 10.3389/fpsyt.2023.1305359

**Published:** 2024-01-08

**Authors:** Yee-Lam E. Chan, Shih-Jen Tsai, Yijuang Chern, Albert C. Yang

**Affiliations:** ^1^Doctoral Degree Program of Translational Medicine, National Yang Ming Chiao Tung University and Academia Sinica, Taipei, Taiwan; ^2^Department of Psychiatry, Cheng Hsin General Hospital, Taipei, Taiwan; ^3^Department of Psychiatry, Taipei Veterans General Hospital, Taipei, Taiwan; ^4^Division of Psychiatry, Faculty of Medicine, National Yang Ming Chiao Tung University, Taipei, Taiwan; ^5^Institute of Biomedical Sciences, Academia Sinica, Taipei, Taiwan; ^6^Institute of Neuroscience, National Yang Ming Chiao Tung University, Taipei, Taiwan; ^7^Institute of Brain Science/Digital Medicine Center, National Yang Ming Chiao Tung University, Taipei, Taiwan; ^8^Department of Medical Research, Taipei Veterans General Hospital, Taipei, Taiwan

**Keywords:** schizophrenia, functional magnetic resonance imaging, hub, network, endophenotypes

## Abstract

**Introduction:**

Pathophysiological etiology of schizophrenia remains unclear due to the heterogeneous nature of its biological and clinical manifestations. Dysfunctional communication among large-scale brain networks and hub nodes have been reported. In this study, an exploratory approach was adopted to evaluate the dysfunctional connectome of brain in schizophrenia.

**Methods:**

Two hundred adult individuals with schizophrenia and 200 healthy controls were recruited from Taipei Veterans General Hospital. All subjects received functional magnetic resonance imaging (fMRI) scanning. Functional connectivity (FC) between parcellated brain regions were obtained. Pair-wise brain regions with significantly different functional connectivity among the two groups were identified and further analyzed for their concurrent ratio of connectomic differences with another solitary brain region (single-FC dysfunction) or dynamically interconnected brain network (network-FC dysfunction).

**Results:**

The right thalamus had the highest number of significantly different pair-wise functional connectivity between schizophrenia and control groups, followed by the left thalamus and the right middle frontal gyrus. For individual brain regions, dysfunctional single-FCs and network-FCs could be found concurrently. Dysfunctional single-FCs distributed extensively in the whole brain of schizophrenia patients, but overlapped in similar groups of brain nodes. A dysfunctional module could be formed, with thalamus being the key dysfunctional hub.

**Discussion:**

The thalamus can be a critical hub in the brain that its dysfunctional connectome with other brain regions is significant in schizophrenia patients. Interconnections between dysfunctional FCs for individual brain regions may provide future guide to identify critical brain pathology associated with schizophrenia.

## Introduction

Precise diagnosis and treatment of schizophrenia have been challenging due to the heterogeneous nature of its biological and clinical manifestations (1). By decomposing the complexity of this neuropsychiatric disorder through endophenotype identification, patients with schizophrenia can be classified into subgroups for further clarification of disorder mechanisms (2). Since the heterogeneity of schizophrenia cannot be sufficiently explained by the presence of a single brain lesion, the dysconnectivity hypothesis suggests that schizophrenia is induced by the abnormal functional integration of brain regions (3, 4). The resting-state functional magnetic resonance imaging (fMRI) is extensively applied to evaluate the alterations of pairwise functional connectivities (FCs) in schizophrenia.

Some fMRI studies have demonstrated the disorganized communication among large-scale brain networks in schizophrenia (5, 6). In a transdiagnostic multimodal meta-analysis of resting-state fMRI, results from 2,069 participants with schizophrenia and 2,106 controls were analyzed. Characteristic brain regions that showed substantial dysconnectivity in schizophrenia included the lateral postcentral cortex, striatum, and thalamus (7). Dong et al. (5) conducted a meta-analysis to search for consistent patterns of network dysfunction, for which 2,115 schizophrenia participants and 2,297 healthy controls from 56 seed-based voxel-wise resting state FC datasets were included. Decreased communication within brain systems critical for salience processing, gating information, emotion processing, somatosensory perceptions, and self-related thoughts was proposed. Additionally, Supekar et al. (6) reported impairment of the dynamic functional interactions of the salience network with the central executive network and the default mode network, using two independent cohorts of resting-state fMRI datasets from 130 schizophrenia participants and 130 matched healthy controls. They suggested that aberrations in salience network circuit dynamics may lead to impaired weighting and attribution of saliency toward external stimuli and internal mental events, and the inappropriate allocation of attentional and working memory resources, thus contributing to the formation of psychosis.

Notable studies have focused on the functional dysregulation of hub nodes within a network. While they are the highly connected and topologically central elements, dysfunction within hub regions can rapidly and massively influence other connected areas (8, 9). Generally, hub nodes of the human connectome that contribute to psychiatric disorders are seated in regions of association cortex and subcortical nuclei (10). Mäntylä et al. (11) compared the similarity of brain signal time courses in each voxel during complex real-life-like stimulation, between 51 patients with first-episode psychosis and 32 community-based controls. Moreover, they measured the hubness and integration of brain areas through calculation of the regional weighted degree centrality across the brain. Significantly weaker inter-subject correlations in widespread brain regions were observed in participants with first-episode psychosis than in controls. Regional magnitude of the between-group difference in inter-subject correlations was associated with hubness, suggesting that the largest inter-subject correlation differences predominate in hub regions.

Some studies tried to capture the within-participant properties to assess the stability of the brain functional connectome. Using publicly available cohorts, Kaufmann et al. (12) gathered functional connectivity profiles of resting state fMRI (rs-fMRI) and two task-fMRI. They attempted in identifying an individual by his connectivity profile in one scanning, from profiles of another scanning among all individuals. The same group reanalyzed task fMRI data sets in individuals with schizophrenia, and calculated the connectome stability in the full brain and subnetworks (13). They concluded that the connectome stability was lower in patients with schizophrenia, and the motor network stability was significantly associated with polygenic risk for schizophrenia. Instead of examining the topology of dysfunctional connectome in schizophrenia, their result may be applied in the search for individualized marker and trajectory of mental health in adolescents. Mennigen et al. (14) adopted a different approach, by investigating the dynamic functional network connectivity in youth with psychosis spectrum syndrome. Intrinsic connectivity networks (ICNs) were identified and assigned to different functional domains based on their anatomical location. ICN-to-ICN connectivity under different dynamic states were compared. Dysconnectivity involving multisensory association cortices was found. The study provided method to identify potential neuroimaging biomarker for youth with psychosis syndrome. However, most individuals will not develop overt psychotic disorder, and longitudinal study will be required. Sun et al. (15) tried to characterize the relationship between intersubject variability of the functional connectome (IVFC) and clinical variables in schizophrenia. The intersubject similarity between any two participants was assessed by Pearson correlation of their functional connectivity profiles. The IVFC was then estimated by subtracting the averaged intersubject similarity from one, to derive a whole-brain IVFC map. Higher IVFC in the bilateral sensorimotor, visual, auditory, and subcortical regions was found in the schizophrenia group, which was further correlated with clinical variables. The result could explain the high clinical heterogeneity of schizophrenia, and might help to classify imaging-derived candidate phenotypes. Yet, the connectomic interaction between brain regions was not the main focus of this study. Mastrandrea et al. (16) evaluated the brain functional architecture through an advanced network analysis of rs-fMRI. Inter-subject variability of the brain functional network correlation matrices was calculated. Percolation analyses was performed, followed with maximum spanning tree filtration to keep the most robust links between nodes. An allometric scaling was then applied to study the topological properties of the functional network. Using a complex analytical pipeline, their result suggested unintegrated change in local connectivity strengths in schizophrenia, causing disruption of local modular organization, homogenization of the patterns and loss of hierarchy for information spread among all brain regions. This echoes with our result that the altered and unbalanced functional connectome in schizophrenia might interfere with the coordination of information or perceptual processing. Yang et al. (17) conducted coactivation pattern (CAP) analysis to investigate rs-fMRI and different frequency sub-bands, for prediction of the diagnostic status of schizophrenia. Yet, the choice of frequency bands division remained to be investigated.

Previous analysis of network dysfunction in schizophrenia might focus on brain networks with presumed importance in psychosis development. Investigations for hub dysfunction in schizophrenia focused on the centrality of individual hub nodes, and their role within structural or functional networks (18). In this study, we adopted an exploratory approach to evaluate the brain dysfunctional connectome in a schizophrenia cohort. All brain regions were analyzed for their concurrent ratio of connectomic differences with another solitary brain region (single-FC dysfunction) or dynamically interconnected brain network (network-FC dysfunction). More single-FC dysfunction can imply that once a brain node becomes dysfunctional, there will be fewer counteracting partners to compensate its function. Meanwhile, we tried to identify potentially dysfunctional hubs and interpreted their interconnections.

## Materials and methods

### Participants

The study cohort comprised 200 individuals with schizophrenia and 200 matched healthy controls aged 20–65 years. Participants were recruited between 2011-January and 2016-December, from the ongoing Taiwan Aging and Mental Illness (TAMI) study conducted in accordance with the Declaration of Helsinki. All schizophrenia participants, recruited from the Taipei Veterans General Hospital, received a diagnosis of schizophrenia according to the diagnostic criteria of the Diagnostic and Statistical Manual of Mental Disorders, Fourth Edition (DSM-IV-TR) by two board-certified psychiatrists. The diagnosis was further validated using the Mini-International Neuropsychiatric Interview (MINI). Furthermore, they received evaluations regarding their positive and negative syndrome scale (PANSS) scores and the chlorpromazine equivalent doses (CPZ equivalents) of the antipsychotics they were taking.

All participants were evaluated to exclude the presence of head trauma history or neurological diseases; lifetime history of alcohol or substance abuse; severe medical illnesses; pacemaker, intracranial clip or any metallic implantation; and current pregnancy or lactation. Healthy controls with personal or family history of psychiatric disorders among first-degree relatives were excluded. Schizophrenia participants with a history of psychiatric disorders other than schizophrenia (e.g., bipolar disorder, schizoaffective disorder, or major depressive disorder) were excluded. All procedures involving human subjects were approved by the Institutional Review Board of Taipei Veterans General Hospital, with written informed consent obtained from all participants.

### Image acquisition and processing

All fMRI scanning procedures were performed at Yang Ming Campus of National Yang Ming Chiao Tung University by using a 3.0T Siemens MRI scanner (Siemens Magnetom Tim Trio, Erlangen, German) equipped with a 12-channel head coil. To avoid motion artifacts generated during the scan, the participant’s head was immobilized with cushions inside the coil after alignment. T2*-weighted images with BOLD contrast were obtained using a gradient echo-planar imaging (EPI) sequence along the anterior commissure-posterior commissure (AC-PC) line (repetition time TR = 2,500 ms, echo time TE = 27 ms, field of view FoV = 200 mm, flip angle = 77°, matrix size = 64 × 64, voxel size = 3.4 × 3.4 × 3.4 mm^3^, slices = 43, total volumes =200). Structural T1 images were acquired using a 3D magnetization-prepared rapid gradient echo sequence (TR = 2,530 ms, TE = 3.5 ms, inversion time TI = 1,100 ms, FoV = 256 mm, flip angle = 7°, matrix size = 256 × 256, voxel size = 1.0 × 1.0 × 1.0 mm^3^, slices = 192).

Resting-state fMRI data were pre-processed and analyzed using SPM12 (Wellcome Department of Imaging Neuroscience, London, United Kingdom), the Resting-State fMRI Data Analysis Toolkit (REST, http://www.restfmri.net) (19) and the DPABI toolbox (version 4.3) (20) implemented in MATLAB (Mathworks Inc., Sherborn, MA). Images were slice-timing corrected (reference: 43) and realigned. Linear co-registration (degrees of freedom:12) was performed between individual T1-weighted (T1w) structural images and the mean functional image after realignment. Linear registration (degrees of freedom:12) was then performed for the transformed structural images, using white-matter boundaries via T1w segmentation. The images were normalized into the standard stereotaxic space of the Montreal Neurological Institute (MNI) EPI template and resampled to a 3 mm cubic voxel. Nuisance covariate regression was performed by the component-based method. Covariates of the BOLD time-series data were regressed out, including the time courses of 6 head motions and their derivatives, the white matter, and the cerebrospinal fluid. Global signal regression was performed so that signal disruption that is widespread and similar in nature over much of the brain is removed. The procedure might help to remove the increased signal correlations between distant voxels induced by motion (21). The registered fMRI data were segmented into 90 brain regions by using an anatomically labeled template (22). For each participant, a representative BOLD time series of each region of the brain was obtained through averaging the BOLD time series of all voxels within a given region. Each regional BOLD time series was corrected for the effect of head movement through regression of the translations and rotations of the head estimated in image realignment.

All participants recruited from the TAMI study exhibited a maximal displacement of less than 1.5 mm at each axis, and an angular motion of less than 1.5° for each axis. Quality control was further performed in our study, using independent *t*-test, to analyze the mean framewise displacement (FD_Power_) across subjects. There was no statistically significant group difference in motion between the schizophrenia and control groups [schizophrenia: mean FD_Power_: 0.16, standard deviation (SD): 0.11; control: mean FD_Power_: 0.15, SD: 0.10. *p*-value: 0.36, 95% confidence interval (95% CI): −0.01 – 0.03]. Thus, no participants have been excluded for excessive head motion in the current study cohort. The first five data points (12.5 s) in all BOLD time series were discarded due to the instability of initial MRI scanning. Temporal band-pass filtering (0.01–0.08 Hz) was performed to reduce the influence of high-frequency noise and low-frequency drift from physiologic confounders.

### Functional connectivity differences between schizophrenia participants and controls

**Figure 1:**
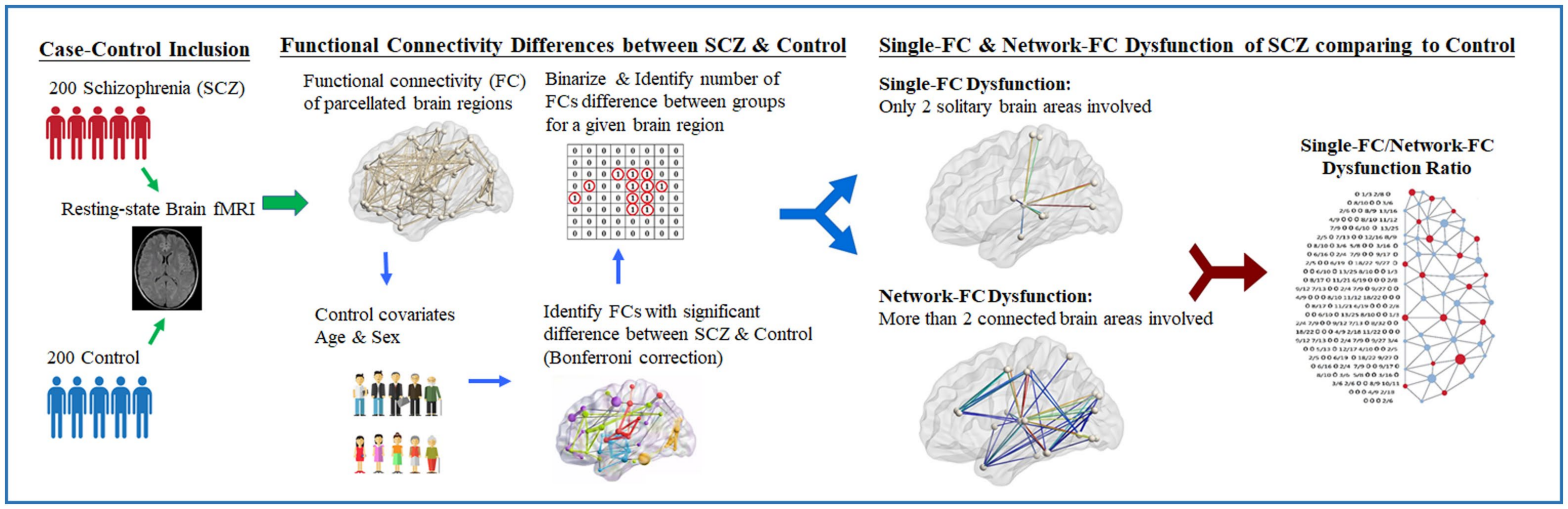
Study design of resting-fMRI analysis in schizophrenia clinical cohort. Illustrated study design flowchart of resting-state fMRI analysis between schizophrenia and control. With the proposed computational method, the single-FC to network-FC dysfunction ratio of brain in schizophrenia can be obtained and mapped onto the AAL-90 atlas.

The flowchart of the research design is presented (Figure 1). Pairwise FCs parcellated according to the automated anatomical labeling atlas (AAL-90) were obtained (22) across 90 brain regions for each subject. By applying a minimum pairwise correlation coefficient (rho[*r*] < −0.23 and *r* > +0.23, *p* < 0.001) for all subjects, pairwise FCs with high significance among individuals were conserved for the following analysis. Between-group differences in pairwise FCs were evaluated using linear regression controlling for age and sex. The significance level of pairwise FCs was corrected using the Bonferroni method based on multiple comparisons among the 90 brain regions (i.e., *p*-value <0.05/4,005 brain regions). Pairwise brain regions with considerably different FCs between the two groups were identified and binarized. The numbers of significantly different FCs for a given brain region were further calculated. The leave-one-subject-out sensitivity analysis was performed between every subject and the remaining 399 individuals. The mean standard error between the true value of the testing data and its predicted value is calculated.

### Single-FC and network-FC dysfunction ratio in schizophrenia

**Figure 2:**
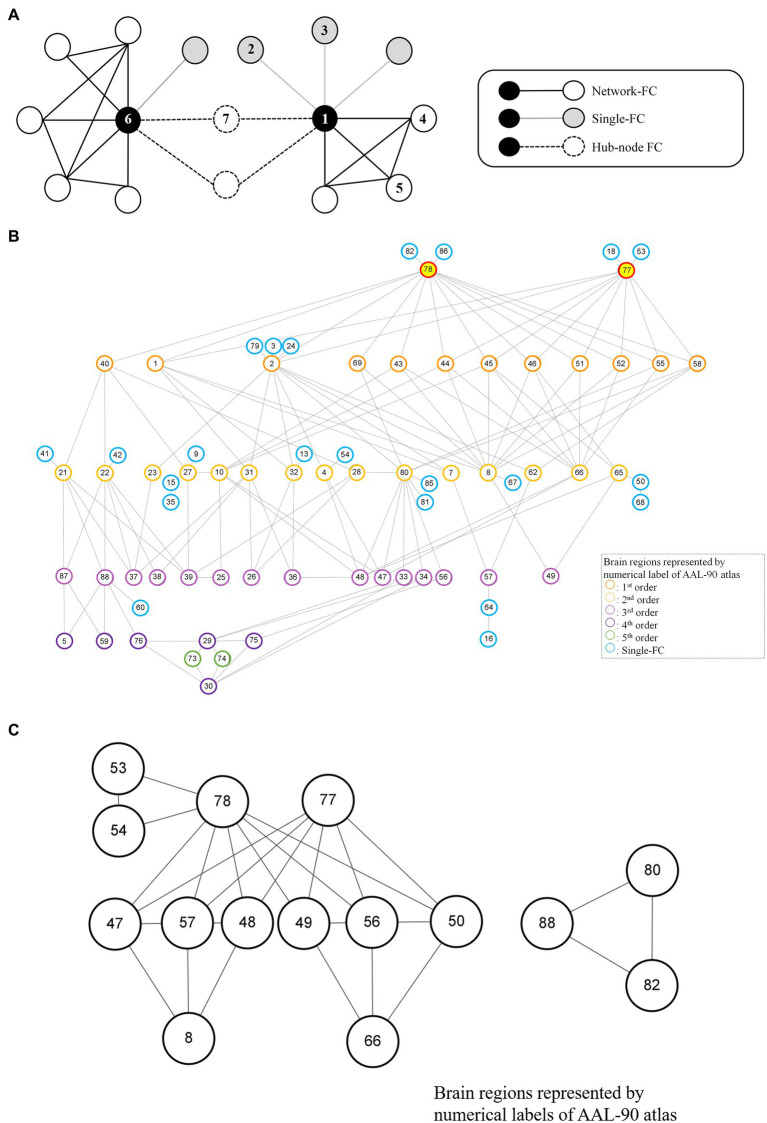
Node-based network and dysfunctional modules in schizophrenia. **(A)** Conceptual representations of network-FC, single-FC and hub-node FC. Brain regions connected to single-FCs with further connections to other brain regions are denoted as hub-node FCs. **(B)** The thalamus module with right thalamus (Label 78) and left thalamus (Label 77) being the key dysfunctional hubs. All single-FCs and hub-node FCs are clustered and arranged in hierarchical orders. **(C)** Dysfunctional modules formed by network-FCs. A larger dysfunctional network was formed between the right thalamus (Label 78) and the left thalamus (Label 77). A smaller dysfunctional network was formed between the right Heschl gyrus (Label 80), the right superior temporal gyrus (Label 82) and the right middle temporal pole (Label 88). Brain regions are represented by numerical label of the AAL-90 atlas (Supplementary Table S1).

For each brain region (e.g., Region 1 network), single-FC dysfunction was defined as the involvement of another solitary brain region showing FC differences between schizophrenia patients and healthy controls (e.g., Region 1–Region 2, Region 1–Region 3), whereas network-FC dysfunction was defined as the involvement of two or more connected brain regions within the same network, showing FC differences between the groups (e.g., Region 1–Region 4–Region 5–Region 1) (Figure 2A). The single-FC and network-FC dysfunction ratios were the number of FC differences involved in single-FC or network-FC, divided by the number of total FC differences for a particular brain region. In other words, the single-FC or network-FC dysfunction ratio for a given brain region became a comparative concept.

### Node-based network and dysfunctional module

A node-based network could be formed between single-FC dysfunctions (Figure 2A). Brain regions connected to another solitary brain nodes were denoted as single-FCs (e.g., Region 1 network: Region 1–Region 2, Region 1–Region 3). Conversely, brain regions indirectly connected via single-FCs to common brain nodes were denoted as hub-node FCs (e.g., Region 1 network: Region 1–Region 7, Region 6 network: Region 6–Region 7). Dysfunctional module was then identified from interconnections between all single-FCs and hub-node FCs by clustering analysis. Visualized plots were created with BrainNet Viewer (23) and Cytoscape (24).

### Correlation with clinical parameters

Local efficiency is a measure of the average efficiency of information transfer between neighbors of a node (25). Higher local efficiency (range: 0–1) indicates higher effectiveness of information transfer among local network. Pearson correlation was conducted between the local efficiency of the identified key dysfunctional hub and the PANSS score. The effect of drug doses was also evaluated. Partial correlations of local efficiency of the thalamus and PANSS scores was performed when directly controlling for CPZ equivalent dose, age, and sex.

## Results

### Participants

Demographic characteristics of schizophrenia participants and normal controls are presented in Table 1. Both groups aged 43.6 years on average, with equally distributed sex ratio. Additionally, no significant differences were observed in the handedness or head motion of participants between the groups. For individuals with schizophrenia, the average disorder onset age was 28.2 years [standard deviation (SD): 10.1] and disorder duration was 15.4 years (SD: 10.9). The mean total PANSS score was 40.4 (SD: 12.3) and CPZ equivalent dose was 403.2 mg/day (SD: 325.4 mg/day).

**Table 1:** Demographic data of schizophrenia participants and healthy controls.

	Control (*n* = 200)	Schizophrenia (*n* = 200)	*p*-value
Age at scan (years, SD)	43.6 (13.4)	43.6 (12.6)	1.00
Sex (male, %)	99 (49.5%)	99 (49.5%)	1.00
Handedness, right	195 (97.5%)	189 (95%)	0.34
Head motion	0.15 (0.10)	0.16 (0.11)	0.36
Framewise displacement (FD_Power_, SD)			
Disorder onset age (years, SD)	—	28.2 (10.1)	
Disorder duration (years, SD)	—	15.4 (10.9)	
PANSS total (SD)	—	40.4 (12.3)	
PANSS positive (SD)	—	9.6 (3.6)	
PANSS negative (SD)	—	9.8 (3.8)	
PANSS general (SD)	—	21.1 (6.1)	
CPZ equivalents (mg/day, SD)	—	403.2 (325.4)	

CPZ equivalents, chlorpromazine equivalents; PANSS, positive and negative syndrome scale; *p*-value, Pearson chi-squared test for nominal variables, independent *t*-test for continuous variables; SD, standard deviation.

### Brain regions with highest number of significantly different FCs between schizophrenia and control groups

**Figure 3:**
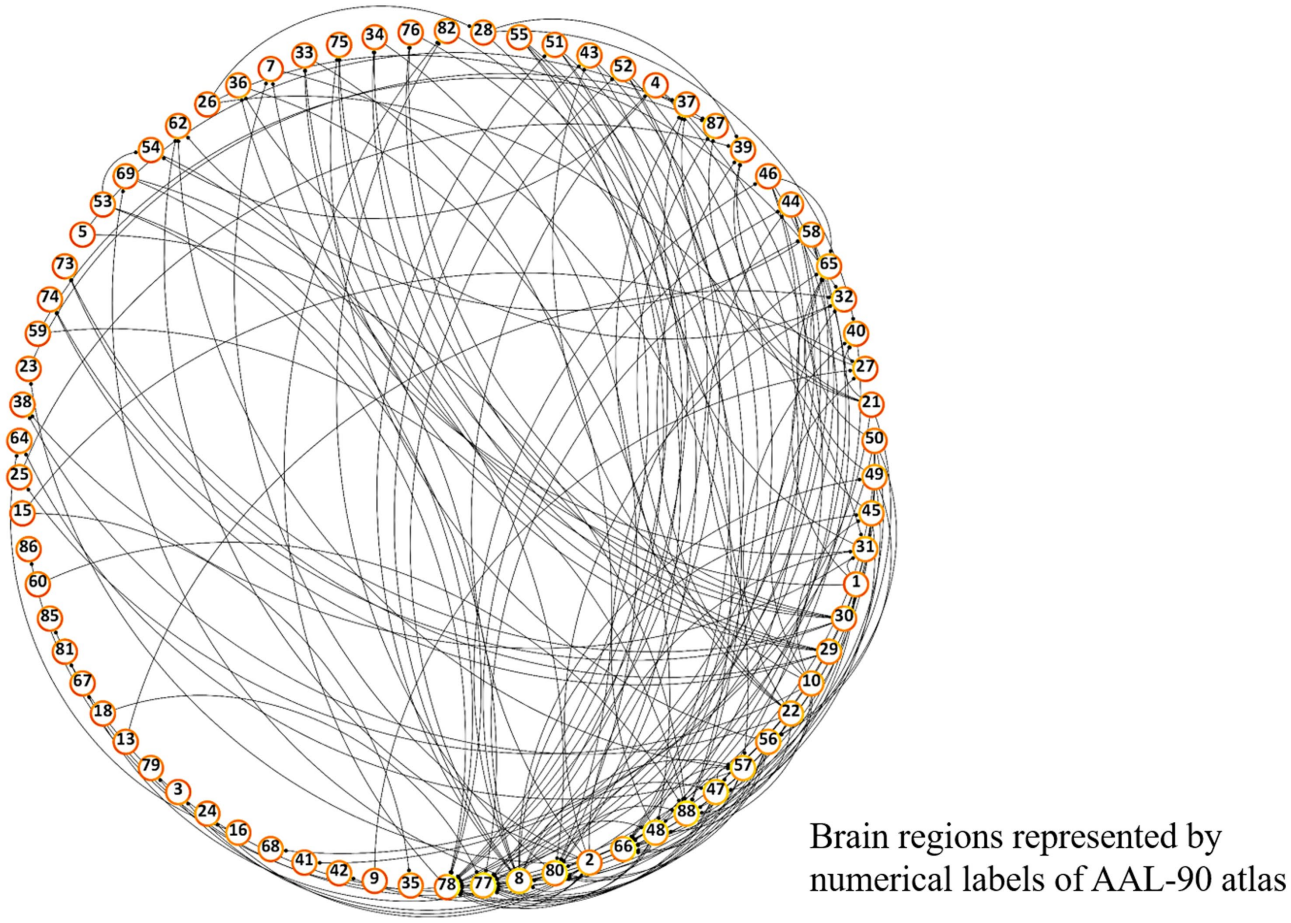
Overall FC dysfunction in schizophrenia patients. Visualized plot of significantly different pairwise functional connectivities between schizophrenia and control groups among AAL-90 brain regions. Brain regions are presented in a clockwise arrangement according to the number of interconnections, and are represented by numerical label of the AAL-90 atlas (Supplementary Table S1).

**Figure 4:**
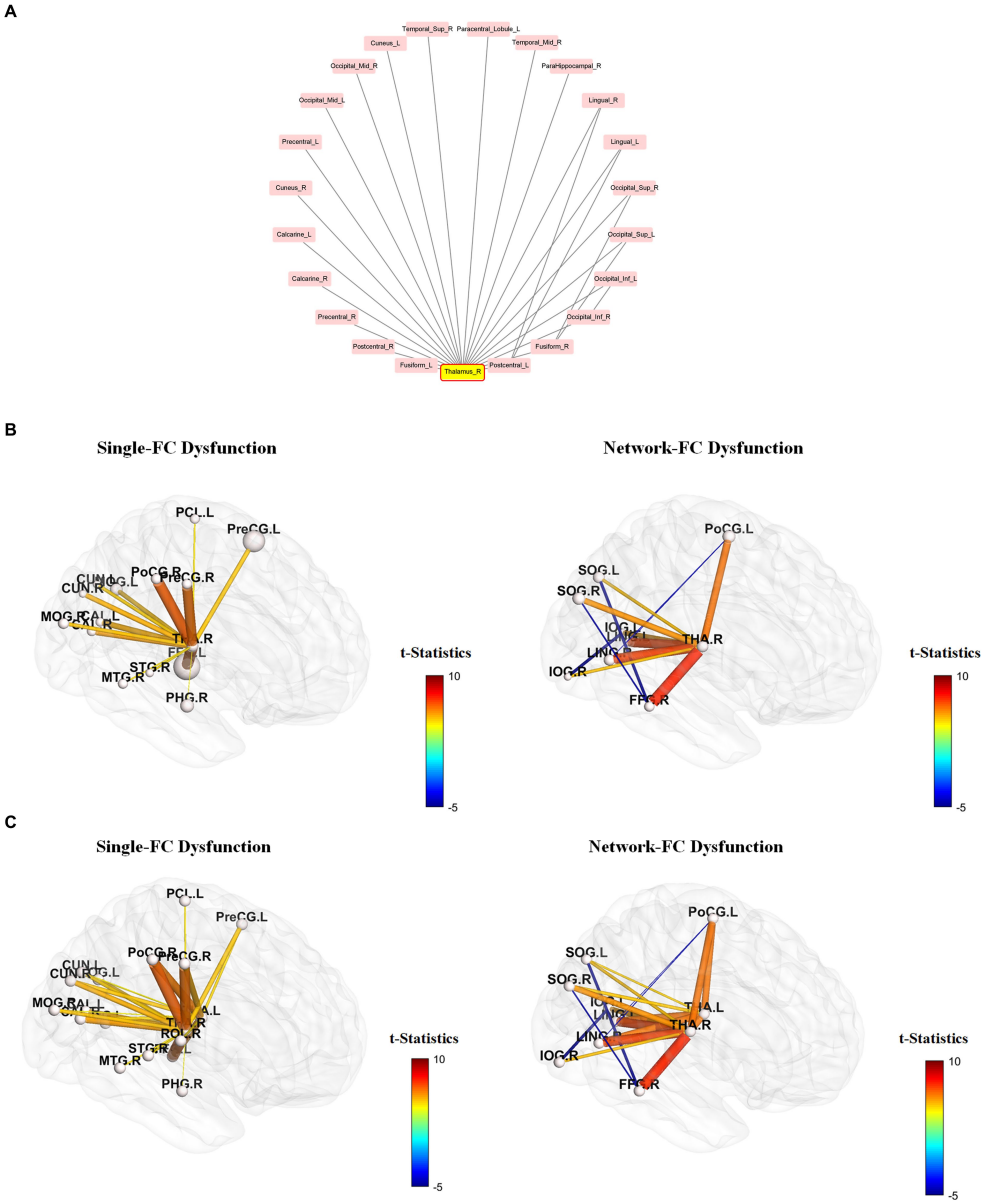
Single-FC dysfunction and network-FC dysfunction within the right-thalamus network. Among the 90 AAL regions, the right thalamus has the highest number of significantly different pair-wise functional connectivity (FC) between schizophrenia and control groups. **(A)** Brain regions connected to the right thalamus are presented in a clockwise arrangement according to the number of interconnections within the right thalamus network. (R, right, L, left, Ant, anterior, Sup, superior, Mid, middle, Inf, inferior, Oper, opercular). **(B,C)** Brain regions connected to the right thalamus **(B)** and bilateral thalamus **(C)** that do not have interconnections within the thalamus network are depicted as single-FC dysfunction regions. Conversely, brain regions connected to the right thalamus **(B)** and bilateral thalamus **(C)** that exhibit additional interconnections within the thalamus network are depicted as network-FC dysfunction regions. Within the thalamus network, FCs of brain regions having direct connections to the thalamus were all increased, but FCs without direct connections to the thalamus were all decreased. Brain regions are represented by abbreviations of the AAL-90 atlas (Supplementary Table S1).

Differences in pairwise FCs between schizophrenia and control groups were analyzed through linear regression, controlling for age and sex (Figure 3). Among the 90 AAL regions, the highest number (*n* = 22) of significantly different pairwise FCs between the groups (Figure 4) was found in the right thalamus, followed by the left thalamus and right middle frontal gyrus (Supplementary Table S1).

Using the right thalamus as an example, brain regions connected to the right thalamus are presented in a clockwise arrangement according to the number of interconnections within the right thalamus network (Figure 4A). Brain regions connected to the right thalamus (Figure 4B) and bilateral thalamus (Figure 4C) that do not have interconnections within the thalamus network are depicted as single-FC dysfunction regions. Conversely, brain regions connected to the right thalamus (Figure 4B) and bilateral thalamus (Figure 4C) that exhibit additional interconnections within the thalamus network are depicted as network-dysfunction regions. The pairwise FC differences between groups are presented based on their *t*-statistics (Figure 4 and Supplementary Table S2). Only connections that reached significance after Bonferroni correction are shown. The range of mean standard error lies between 2.37 * e^−29^ and 3.28 * e^−28^ in the leave-one-subject-out sensitivity analysis, indicating that the heterogeneity of FC signal between subjects is negligible. Within the thalamus network, FCs of brain regions having direct connections to the thalamus were all increased, but FCs without direct connections to the thalamus were all decreased.

### Single-FC and network-FC dysfunction ratios in schizophrenia

**Figure 5:**
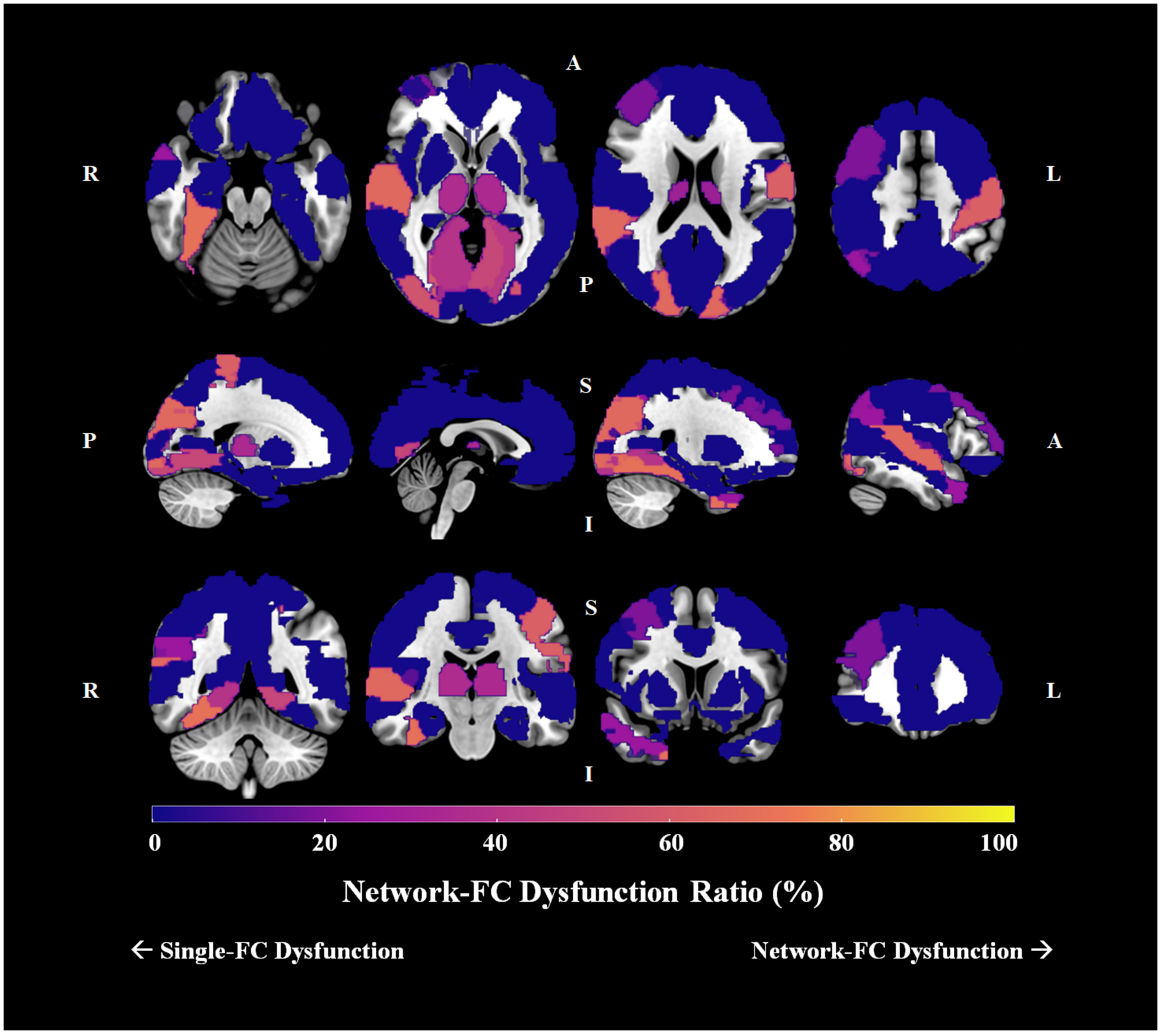
Network-FC dysfunction ratio among 90 brain regions in schizophrenia. The single-FC or network-FC dysfunction ratio for a given brain region is a comparative concept. Hot regions (bright yellow) in the figure represent areas with higher network-FC dysfunction ratio and lower single-FC dysfunction ratio; whereas cold regions (dark purple) represent areas with lower network-FC dysfunction ratio but higher single-FC dysfunction ratio. (Orientation of brain slices: L, left, R, right, A, anterior, P, posterior, S, superior, I, inferior).

Group differences in pairwise FCs were analyzed among the 90 AAL brain regions. For schizophrenia patients, 16 brain regions had no significant connection difference at all, 16 had only one connection difference, and the remaining 58 had more than one connection differences. For the 58 brain regions with more than one connection differences, we determined the single-FC and network-FC dysfunction ratio by using the aforementioned method (Supplementary Table S3). In total, 43 brain regions showed absolute single-FC dysfunction, 7 showed >50% single-FC dysfunction, 1 showed equal ratio between single-FC dysfunction and network-FC dysfunction, and 7 showed >50% network-FC dysfunction. In other words, 55.6% of whole brain regions showed a ratio favoring single-FC dysfunction, while 7.8% of the brain regions showed a ratio suggesting network-FC dysfunction. Using the right thalamus as an example, 14 brain regions connected to the right thalamus showed single-FC dysfunction and 8 showed network-FC dysfunction. Thus, the right thalamus showed ratios of 63.6% single-FC dysfunction and 36.4% network-FC dysfunction. The network dysfunction ratio ranging from 0% to 100% is then visualized and plotted onto a brain atlas according to the corresponding color intensity (Figure 5). Hot regions in the figure represent regions with more network-FC dysfunction while the cold regions represent regions with more single-FC dysfunction (Figure 5).

### Node-based network and dysfunctional module

Although more brain regions were involved in single-FC dysfunction, FCs for these solitary brain regions overlapped extensively and a network structure was formed among the hub-node FCs (Figures 2A, 4C). All single-FCs and hub-node FCs were clustered and arranged in hierarchical orders. A dysfunctional module was then identified, with bilateral thalamus (Labels 77, 78) being the key dysfunctional hub (Figure 2B). In the thalamus module, several highly interconnected hub-node FCs were found, including bilateral precental gyrus (Labels 1, 2), cuneus (Labels 45, 46) and occipital region (Labels 43, 44, 51, 52, 55) in the level 1 nodes; right middle frontal gyrus (Label 8), right angular gyrus (Label 66), right Heschl gyrus (Label 80) and bilateral olfactory cortex (Labels 21, 22) in the level 2 nodes.

Two sets of dysfunctional modules were formed by network-FCs (Figure 2C), with one being the dysfunctional thalamus network (Labels 77, 78). Another small set of dysfunctional network-FCs formed between the right Heschl gyrus (Label 80), the right superior temporal gyrus (Label 82) and the right middle temporal pole (Label 88).

### Correlation with clinical parameters

PANSS items that capture the key symptoms of schizophrenia, namely hallucinatory behavior (P3) (left thalamus: *r* = −0.15, *p*-value = 0.034; right thalamus: *r* = −0.17, *p*-value = 0.021) and suspiciousness/persecution (P6) (left thalamus: *r* = −0.17, *p*-value = 0.016; right thalamus: *r* = −0.18, *p*-value = 0.015) were significantly and inversely associated with the local efficiency of the thalamus.

To examine if the study findings could be driven by medication, partial correlations of local efficiency of the thalamus and PANSS scores was performed when directly controlling for CPZ equivalent dose, age, and sex. A statistically significant and inverse correlation was still found between local efficiency of the thalamus and positive scale of PANSS (PANSS-P) (right thalamus: *r* = −0.15, *p*-value = 0.045), hallucinatory behavior (P3) (left thalamus: *r* = −0.17, *p*-value = 0.018; right thalamus: *r* = −0.19, *p*-value = 0.012), and suspiciousness/persecution (P6) (left thalamus: *r* = −0.19, *p*-value = 0.012; right thalamus: *r* = −0.20, *p*-value = 0.007).

## Discussion

This study investigated differences in FCs between patients with schizophrenia and healthy controls based on resting-state fMRI data. Connectivity dysfunction between the thalamus and its connected brain regions could play a crucial role in schizophrenia pathophysiology. The single-FC to network-FC dysfunction ratios of individual brain regions were further determined and visualized using an AAL atlas. 55.6% of whole brain regions showed a ratio favoring single-FC dysfunction, while 7.8% of the brain regions showed a ratio suggesting network-FC dysfunction. In addition, interconnections between all single-FCs and hub-node FCs were analyzed by clustering analysis. Many of the hub nodes were impaired and highly overlapped, which were further classified into a dysfunctional module with bilateral thalamus being the key dysfunctional hub.

### Role of thalamus in schizophrenia

Considerable evidence has shown that thalamocortical FC could be dysregulated in schizophrenia (26, 27). In coherence with this speculation, the right and left thalamus were found to have the highest numbers of different interconnected FCs among schizophrenia participants in our study. Being a heterogeneous structure composed of multiple nuclei with distinct inputs and cortical outputs, the thalamus is considered a relay center within the brain that transmits peripheral information to the cortex (28). It carries out diverse functions, ranging from the regulation of cognition, memory, sensory signals and mood, to the control of language functions, motor signals, and even consciousness (29). Furthermore, increasing studies have indicated that the thalamus possesses gating properties to modulate the effectiveness of input signals (30). Given its relay and gating nature, alterations in varying magnitudes of FC between the thalamus and different brain regions might be one of the keys that shape the diversity of clinical phenotypes in schizophrenia. Since the thalamus can be subdivided into multiple distinct nuclei with different anatomical connections to various cortical regions, Hua et al. conducted a fMRI study by using ultra-high magnetic field (7.0 Tesla) to examine the seed-based FC between subthalamic regions and other brain areas in individuals with schizophrenia (31). They reported impairment of the thalamic connectivity to the prefrontal cortex and the cerebellum, but enhanced thalamic connectivity to the motor/sensory cortex in schizophrenia. However, their study had a fundamental limitation of a small sample size with only 14 schizophrenia patients and 14 controls. Thus, sensitivity was insufficient to correlate individual seed region to any clinical marker.

Our results indicate substantial dysfunctional connectome between the thalamus and its connected brain regions, highlighting the salient role of the thalamus in schizophrenia. A close examination of the right thalamus network (Figure 4B) revealed increased FCs between brain regions with direct connections to the right thalamus in schizophrenia. Furthermore, FCs between regions without direct connections to the right thalamus were all decreased. Similar findings were also observed when the left thalamus was taken into account (Figure 4C), but there were different patterns of dysfunctional connectome for other brain nodes. This imbalance of FC distribution possibly reflected the sensory processing dysfunction and overload among schizophrenia participants (32). In fact, such observation is compatible with the neuroanatomical properties of the thalamus, which is mainly composed of excitatory glutamatergic relay cells, inhibitory GABAergic interneurons, and reticular cells (33). Our results revealed increased functional coupling between the thalamus and sensorimotor cortices in schizophrenia, which is consistent with previous reports (34, 35). Moreover, FCs between the thalamus and brain areas driving different perceptions increased. For instance, the occipital gyrus (Labels 51, 52), calcarine cortex (Labels 43, 44), cuneus (Labels 45, 46), fusiform gyrus (Labels 55, 56) and lingual gyrus (Labels 47, 48) that govern visual functions, and the temporal gyrus (Labels 81, 82, 85, 86, 87, 88) that regulate auditory functions were involved.

Apart from the dysfunctional thalamus network (Labels 77, 78), there was only a small set of dysfunctional network-FC forming between the right Heschl gyrus (Label 80), the right superior temporal gyrus (Label 82) and right middle temporal pole (Label 88). This network might have influence over auditory functions, face recognition, autobiographic memory, semantic and even socio-emotional processing (36, 37).

### Role of other critical brain regions in schizophrenia

Other than the thalamus, dysfunctional FCs distributed across different lobes of the brain in schizophrenia patients. Brain regions with significant FCs differences among schizophrenia (SCZ) and control were found in the frontal lobe, the parietal lobe, the temporal lobe, and the occipital lobe (Supplementary Tables S1, S3).

Prefrontal cortex dysfunction has been reported to induce cognitive control disturbances in schizophrenia (38). The functional networks between the fronto-parietal lobes and the cingulo-opercular regions are hypothesized to drive executive functioning via top-down control (39). In a task-fMRI study, schizophrenia patients and matched controls were asked to perform a working memory binding task. The patients showed abnormal functioning between the ventrolateral prefrontal cortex (VLPFC) and the posterior parietal cortex, leading to impairment in attention and the use of strategies to memorize correctly. Besides, greater activation was found in the left thalamus for schizophrenia patients during the early maintenance period (40).

In a randomized sham-controlled transcranial direct current stimulation (tDCS) study, resting-state functional connectivity (rs-FC) of the left temporo-parietal junction (TPJ) in schizophrenia patients was investigated. Along with reduced rs-FC of the left TPJ with the left anterior insula and the right inferior frontal gyrus after active tDCS, auditory hallucination was also significantly reduced. The result indicated potential for reduction of schizophrenia symptoms, through the modulation of dysfunctional brain network (41).

Governing visual processing, damage in the occipital lobe is associated with visual hallucination in schizophrenia. Using fMRI, a recent study demonstrated that the primary visual cortex was dissociated from other vision-related network during visual hallucination in psychotic patients (42). The occipital lobe may work together with the thalamus to process emotional and social information (43).

### Single-FC dysfunction and network-FC dysfunction in schizophrenia

In this study, we found single-FC dysfunctions widely distributed in the whole brain of schizophrenia patients during resting state, and our results were not generated from any presumptions regarding the anatomy or cognitive processes. Despite extensively found single-FC dysfunctions, FCs for these solitary brain regions overlapped in similar groups of brain nodes. For example, dysfunctional FCs were not formed directly between the right thalamus (Label 78) and left thalamus (Label 77), but their dysfunctional single-FCs overlapped at brain areas controlling the sensorimotor, visual and auditory functions (Figure 2B). It also implied that both the right and left thalamus work in synchronized way although this key dysfunctional hub formed dysfunctional connectomes with multiple brain areas in schizophrenia. Importantly, local efficiency of the thalamus was significantly and inversely associated with positive scale of PANSS, and key symptoms of schizophrenia (i.e., hallucinatory behavior and suspiciousness/persecution). The result indicates that the correlation between local efficiency of the thalamus and PANSS score was not driven by medication dose, age or sex.

Interestingly, the dysfunctional module identified in our analytic approach revealed findings compatible to the neuroanatomical positions and circuitry. For instance, dysfunctional FCs were formed at bilateral olfactory cortex (Labels 21, 22), interconnected with bilateral amygdala (Labels 41, 42), temporal pole (Labels 87, 88) and hippocampus (Labels 37, 38). It reflected the distinct functional connectivity profiles of the olfactory system, which bypassed the thalamus apart from all other sensory systems (44). The affective network which included the anterior cingulate cortex (Labels 31, 32), amygdala (Labels 41, 42), orbitofrontal cortex (Labels 9, 10, 15) and medial prefrontal cortex (Label 23), was found hidden in the 2nd order brain nodes in our dysfunctional module. This network might play an important role in the suppression of impulsive or aggressive behaviors (45). Close interconnections between dysfunctional FCs could also be found between the striatum (Labels 73, 74, 75, 76), insula (Labels 29, 30) and cingulate cortex (Labels 33, 34). These brain regions might take part in the salience network and ventral attention network (46, 47). Normal functioning of these networks help to direct attention to salient sensory stimulations, whereas onset of psychosis has been attributed to disruption of either network (48).

A meta-analysis that included more than 5,000 patients with schizophrenia and 5,000 controls from 314 task-based fMRI studies mapped the coordinates of significant between-group differences onto a previously published functional brain network (49). Although altered functional activation was observed in various brain regions in schizophrenia, peripheral nodes tended to be task-specific and under-activated, whereas the centrally located network nodes were usually over-activated but less specific in function. Their study design inevitably involved participants and cognitive tasks with great variations. Besides, studies without significant between-group differences were excluded from the analysis. Yet, the study still provided an important insight that schizophrenia could be related to sets of abnormally activated hubs according to the task performed, instead of solitary hub dysfunction. Our study revealed compatible results for schizophrenia patients with their brains under resting-state.

Collectively, results of this study indicated high interconnections and network structure between sets of key dysfunctional hubs in schizophrenia. Further studies are needed to identify the causal relationship and alterations in connectivity among these hub nodes to develop a network-based treatment strategy using brain modulation tools. With the robust development of noninvasive deep brain stimulation, focal stimulation of neurons at the subcortical depth without affecting the overlying cortex was proposed (50). Our study will assist the selection of appropriate brain regions as targets of interventions using novel treatment modalities.

## Limitations

Certain study limitations should be considered. First, our participants with schizophrenia were treated with antipsychotics and the drug effect on regional BOLD signal could not be omitted. Still, drug dosages were not significantly associated to the local efficiency of key dysfunctional hub identified in this study. Furthermore, our results demonstrated fundamental differences in the brain functional connectome of patients with schizophrenia, even that our patient cohort showed only mild symptom severity under treatment. Second, our analytical approach might be biased towards identifying more single-FC dysfunctions than network-FC dysfunctions, since single-FC dysfunction only required differences in two interconnected regions to reach statistical significance. For weakly connected brain regions, the bias towards identifying more single-FC dysfunctions is further increased. The single-FC and network-FC dysfunction ratio could also be affected by the preset *p*-value. Specifically, before the correction of *p*-value for multiple comparisons, more interconnected pairwise FCs were different between patients with schizophrenia and controls, which in turn increased the ratio of network-FC dysfunction. However, by using the most stringent criteria with minimum pairwise correlation coefficient for individual FCs, and Bonferroni correction for groupwise comparison, we ensured the significance level of our findings. Moreover, we further elaborated the highly interconnected nature between brain nodes that formed a dysfunctional module in schizophrenia.

## Conclusion

An exploratory approach was adopted to analyze significant dysfunctional FCs across the whole brain in schizophrenia. This study revealed that the thalamus is central to dysfunction of brain connectomes in schizophrenia, suggesting its principal role in schizophrenia pathophysiology through dysfunctional connectome with other brain regions. For individual brain regions, dysfunctional single-FCs and network-FCs could be found concurrently. In combination with the dysfunctional module which demonstrate the interconnections between single-FCs, our study results may represent distinct signature of dysfunctional connectome for individual brain regions in schizophrenia. It also provides insight and clues for the establishment of network-based treatment strategies using non-invasive brain stimulations.

## Data availability statement

The original contributions presented in the study are included in the article/Supplementary material, further inquiries can be directed to the corresponding author.

## Ethics statement

The studies involving humans were approved by Institutional Review Board of Taipei Veterans General Hospital, Taiwan. The studies were conducted in accordance with the local legislation and institutional requirements. The participants provided their written informed consent to participate in this study.

## Author contributions

Y-LEC: Conceptualization, Formal analysis, Investigation, Methodology, Visualization, Writing – original draft, Writing – review & editing. S-JT: Data curation, Funding acquisition, Project administration, Supervision, Validation, Writing – review & editing. YC: Funding acquisition, Supervision, Validation, Writing – review & editing. ACY: Conceptualization, Data curation, Formal analysis, Funding acquisition, Investigation, Methodology, Project administration, Resources, Supervision, Writing – original draft, Writing – review & editing.
