# Supplementary material for: Exploring the role of hub and network dysfunction in brain connectomes of schizophrenia using functional magnetic resonance imaging
Source: Front Psychiatry. 2024 Jan 8;14:1305359. doi: 10.3389/fpsyt.2023.1305359 (PMC10800602; doi:10.3389/fpsyt.2023.1305359)
Supplement: Supplementary file 1 [file Table_1.DOCX]

| **Supplementary Table 1. Number of significantly different pairwise functional connectivities between schizophrenia and control groups among AAL-90 brain regions** | | | |
| --- | --- | --- | --- |
| Labels | Brain Regions | Abbreviations | Number of FCs Differences Between Groups |
| 78 | Thalamus_R | THA.R | 22 |
| 77 | Thalamus_L | THA.L | 17 |
| 8 | Frontal_Mid_R | MFG.R | 15 |
| 80 | Heschl_R | HES.R | 14 |
| 2 | Precentral_R | PreCG.R | 13 |
| 66 | Angular_R | ANG.R | 12 |
| 48 | Lingual_R | LING.R | 10 |
| 47 | Lingual_L | LING.L | 8 |
| 57 | Postcentral_L | PoCG.L | 8 |
| 88 | Temporal_Pole_Mid_R | TPOmid.R | 8 |
| 10 | Frontal_Mid_Orb_R | ORBmid.R | 7 |
| 22 | Olfactory_R | OLF.R | 7 |
| 56 | Fusiform_R | FFG.R | 7 |
| 1 | Precentral_L | PreCG.L | 6 |
| 21 | Olfactory_L | OLF.L | 6 |
| 29 | Insula_L | INS.L | 6 |
| 30 | Insula_R | INS.R | 6 |
| 31 | Cingulum_Ant_L | ACG.L | 6 |
| 45 | Cuneus_L | CUN.L | 6 |
| 49 | Occipital_Sup_L | SOG.L | 6 |
| 50 | Occipital_Sup_R | SOG.R | 6 |
| 27 | Rectus_L | REC.L | 5 |
| 32 | Cingulum_Ant_R | ACG.R | 5 |
| 39 | ParaHippocampal_L | PHG.L | 5 |
| 40 | ParaHippocampal_R | PHG.R | 5 |
| 44 | Calcarine_R | CAL.R | 5 |
| 46 | Cuneus_R | CUN.R | 5 |
| 58 | Postcentral_R | PoCG.R | 5 |
| 65 | Angular_L | ANG.L | 5 |
| 4 | Frontal_Sup_R | SFGdor.R | 4 |
| 28 | Rectus_R | REC.R | 4 |
| 37 | Hippocampus_L | HIP.L | 4 |
| 43 | Calcarine_L | CAL.L | 4 |
| 51 | Occipital_Mid_L | MOG.L | 4 |
| 52 | Occipital_Mid_R | MOG.R | 4 |
| 55 | Fusiform_L | FFG.L | 4 |
| 87 | Temporal_Pole_Mid_L | TPOmid.L | 4 |
| 7 | Frontal_Mid_L | MFG.L | 3 |
| 26 | Frontal_Med_Orb_R | ORBsupmed.R | 3 |
| 33 | Cingulum_Mid_L | DCG.L | 3 |
| 34 | Cingulum_Mid_R | DCG.R | 3 |
| 36 | Cingulum_Post_R | PCG.R | 3 |
| 53 | Occipital_Inf_L | IOG.L | 3 |
| 54 | Occipital_Inf_R | IOG.R | 3 |
| 62 | Parietal_Inf_R | IPL.R | 3 |
| 69 | Paracentral_Lobule_L | PCL.L | 3 |
| 75 | Pallidum_L | PAL.L | 3 |
| 76 | Pallidum_R | PAL.R | 3 |
| 82 | Temporal_Sup_R | STG.R | 3 |
| 5 | Frontal_Sup_Orb_L | ORBsup.L | 2 |
| 15 | Frontal_Inf_Orb_L | ORBinf.L | 2 |
| 23 | Frontal_Sup_Medial_L | SFGmed.L | 2 |
| 25 | Frontal_Med_Orb_L | ORBsupmed.L | 2 |
| 38 | Hippocampus_R | HIP.R | 2 |
| 59 | Parietal_Sup_L | SPG.L | 2 |
| 64 | SupraMarginal_R | SMG.R | 2 |
| 73 | Putamen_L | PUT.L | 2 |
| 74 | Putamen_R | PUT.R | 2 |
| 3 | Frontal_Sup_L | SFGdor.L | 1 |
| 9 | Frontal_Mid_Orb_L | ORBmid.L | 1 |
| 13 | Frontal_Inf_Tri_L | IFGtriang.L | 1 |
| 16 | Frontal_Inf_Orb_R | ORBinf.R | 1 |
| 18 | Rolandic_Oper_R | ROL.R | 1 |
| 24 | Frontal_Sup_Medial_R | SFGmed.R | 1 |
| 35 | Cingulum_Post_L | PCG.L | 1 |
| 41 | Amygdala_L | AMYG.L | 1 |
| 42 | Amygdala_R | AMYG.R | 1 |
| 60 | Parietal_Sup_R | SPG.R | 1 |
| 67 | Precuneus_L | PCUN.L | 1 |
| 68 | Precuneus_R | PCUN.R | 1 |
| 79 | Heschl_L | HES.L | 1 |
| 81 | Temporal_Sup_L | STG.L | 1 |
| 85 | Temporal_Mid_L | MTG.L | 1 |
| 86 | Temporal_Mid_R | MTG.R | 1 |
| 6 | Frontal_Sup_Orb_R | ORBsup.R | 0 |
| 11 | Frontal_Inf_Oper_L | IFGoperc.L | 0 |
| 12 | Frontal_Inf_Oper_R | IFGoperc.R | 0 |
| 14 | Frontal_Inf_Tri_R | IFGtriang.R | 0 |
| 17 | Rolandic_Oper_L | ROL.L | 0 |
| 19 | Supp_Motor_Area_L | SMA.L | 0 |
| 20 | Supp_Motor_Area_R | SMA.R | 0 |
| 61 | Parietal_Inf_L | IPL.L | 0 |
| 63 | SupraMarginal_L | SMG.L | 0 |
| 70 | Paracentral_Lobule_R | PCL.R | 0 |
| 71 | Caudate_L | CAU.L | 0 |
| 72 | Caudate_R | CAU.R | 0 |
| 83 | Temporal_Pole_Sup_L | TPOsup.L | 0 |
| 84 | Temporal_Pole_Sup_R | TPOsup.R | 0 |
| 89 | Temporal_Inf_L | ITG.L | 0 |
| 90 | Temporal_Inf_R | ITG.R | 0 |

| R: Right, L: Left, Ant: Anterior, Inf: Inferior, Med: Medial, Mid: Middle, Oper: Opercular, Orb: Orbital, Post: Posterior, Sup: Superior, Tri: Triangular part |
| --- |
