# Supplementary material for: Exploring the role of hub and network dysfunction in brain connectomes of schizophrenia using functional magnetic resonance imaging
Source: Front Psychiatry. 2024 Jan 8;14:1305359. doi: 10.3389/fpsyt.2023.1305359 (PMC10800602; doi:10.3389/fpsyt.2023.1305359)
Supplement: Supplementary file 2 [file Table_2.DOCX]

| **Supplementary Table 2. Functional connectivity (FC) between thalamus and other brain regions with significant differences among schizophrenia (SCZ) and control** | | | |
| --- | --- | --- | --- |
| Region 1 (R1) | Region 2 (R2) | t-Statistics of FC  R1 to R2 | p-value of FC  R1 to R2 |
| Fusiform_R | Thalamus_R | 7.68 | 1.22E-13 |
| Lingual_R | Thalamus_R | 7.16 | 4.05E-12 |
| Lingual_L | Thalamus_R | 7.07 | 7.07E-12 |
| Fusiform_L | Thalamus_R | 6.70 | 7.16E-11 |
| Postcentral_R | Thalamus_R | 6.63 | 1.08E-10 |
| Lingual_L | Thalamus_L | 6.54 | 1.90E-10 |
| Postcentral_L | Thalamus_R | 6.50 | 2.39E-10 |
| Fusiform_R | Thalamus_L | 6.48 | 2.80E-10 |
| Precentral_R | Thalamus_R | 6.44 | 3.40E-10 |
| Lingual_R | Thalamus_L | 6.42 | 3.99E-10 |
| Postcentral_L | Thalamus_L | 6.32 | 6.91E-10 |
| Postcentral_R | Thalamus_L | 6.10 | 2.57E-09 |
| Occipital_Sup_R | Thalamus_R | 6.04 | 3.51E-09 |
| Fusiform_L | Thalamus_L | 5.86 | 9.59E-09 |
| Calcarine_R | Thalamus_R | 5.77 | 1.58E-08 |
| Occipital_Inf_L | Thalamus_R | 5.72 | 2.07E-08 |
| Precentral_R | Thalamus_L | 5.59 | 4.21E-08 |
| Calcarine_L | Thalamus_R | 5.56 | 4.93E-08 |
| Cuneus_R | Thalamus_R | 5.42 | 1.03E-07 |
| Precentral_L | Thalamus_R | 5.32 | 1.74E-07 |
| Occipital_Sup_R | Thalamus_L | 5.30 | 1.97E-07 |
| Occipital_Mid_L | Thalamus_R | 5.28 | 2.15E-07 |
| Occipital_Sup_L | Thalamus_R | 5.22 | 2.85E-07 |
| Cuneus_R | Thalamus_L | 5.21 | 3.03E-07 |
| Occipital_Inf_R | Thalamus_R | 5.19 | 3.36E-07 |
| Occipital_Mid_R | Thalamus_R | 5.16 | 3.89E-07 |
| Cuneus_L | Thalamus_R | 5.08 | 5.72E-07 |
| Calcarine_R | Thalamus_L | 5.05 | 6.72E-07 |
| Occipital_Inf_L | Thalamus_L | 4.95 | 1.12E-06 |
| Occipital_Sup_L | Thalamus_L | 4.92 | 1.26E-06 |
| Precentral_L | Thalamus_L | 4.90 | 1.40E-06 |
| Rolandic_Oper_R | Thalamus_L | 4.85 | 1.81E-06 |
| Temporal_Sup_R | Thalamus_R | 4.79 | 2.32E-06 |
| Paracentral_Lobule_L | Thalamus_R | 4.70 | 3.58E-06 |
| Temporal_Mid_R | Thalamus_R | 4.69 | 3.81E-06 |
| Occipital_Mid_L | Thalamus_L | 4.60 | 5.68E-06 |
| Cuneus_L | Thalamus_L | 4.59 | 5.99E-06 |
| Occipital_Mid_R | Thalamus_L | 4.58 | 6.12E-06 |
| ParaHippocampal_R | Thalamus_R | 4.51 | 8.63E-06 |
| Lingual_L | Postcentral_L | -4.46 | 1.08E-05 |
| Lingual_R | Postcentral_L | -4.53 | 7.75E-06 |
| Occipital_Sup_R | Fusiform_R | -4.73 | 3.09E-06 |
| Occipital_Sup_L | Fusiform_R | -5.12 | 4.78E-07 |
| Occipital_Inf_L | Occipital_Inf_R | -5.15 | 4.10E-07 |
| R: Right, L: Left, Inf: Inferior, Mid: Middle, Oper: Opercular, Sup: Superior | | | |
