# Supplementary material for: Exploring the role of hub and network dysfunction in brain connectomes of schizophrenia using functional magnetic resonance imaging
Source: Front Psychiatry. 2024 Jan 8;14:1305359. doi: 10.3389/fpsyt.2023.1305359 (PMC10800602; doi:10.3389/fpsyt.2023.1305359)
Supplement: Supplementary file 3 [file Table_3.DOCX]

| **Supplementary Table 3. Single-FC and network-FC dysfunction ratio of brain regions with significant differences among schizophrenia (SCZ) and control** | | | |
| --- | --- | --- | --- |
| Brain Regions | Number of FCs Differences in Total | Single-FC Dysfunction Ratio | Network-FC Dysfunction Ratio |
| Thalamus_R | 22 | 0.64 | 0.36 |
| Thalamus_L | 17 | 0.65 | 0.35 |
| Frontal_Mid_R | 15 | 0.80 | 0.20 |
| Heschl_R | 14 | 0.86 | 0.14 |
| Precentral_R | 13 | 1.00 | 0.00 |
| Angular_R | 12 | 0.75 | 0.25 |
| Lingual_R | 10 | 0.60 | 0.40 |
| Postcentral_L | 8 | 0.38 | 0.63 |
| Lingual_L | 8 | 0.50 | 0.50 |
| Temporal_Pole_Mid_R | 8 | 0.75 | 0.25 |
| Fusiform_R | 7 | 0.29 | 0.71 |
| Frontal_Mid_Orb_R | 7 | 1.00 | 0.00 |
| Olfactory_R | 7 | 1.00 | 0.00 |
| Occipital_Sup_L | 6 | 0.33 | 0.67 |
| Occipital_Sup_R | 6 | 0.33 | 0.67 |
| Precentral_L | 6 | 1.00 | 0.00 |
| Olfactory_L | 6 | 1.00 | 0.00 |
| Insula_L | 6 | 1.00 | 0.00 |
| Insula_R | 6 | 1.00 | 0.00 |
| Cingulum_Ant_L | 6 | 1.00 | 0.00 |
| Cuneus_L | 6 | 1.00 | 0.00 |
| Rectus_L | 5 | 1.00 | 0.00 |
| Cingulum_Ant_R | 5 | 1.00 | 0.00 |
| ParaHippocampal_L | 5 | 1.00 | 0.00 |
| ParaHippocampal_R | 5 | 1.00 | 0.00 |
| Calcarine_R | 5 | 1.00 | 0.00 |
| Cuneus_R | 5 | 1.00 | 0.00 |
| Postcentral_R | 5 | 1.00 | 0.00 |
| Angular_L | 5 | 1.00 | 0.00 |
| Frontal_Sup_R | 4 | 1.00 | 0.00 |
| Rectus_R | 4 | 1.00 | 0.00 |
| Hippocampus_L | 4 | 1.00 | 0.00 |
| Calcarine_L | 4 | 1.00 | 0.00 |
| Occipital_Mid_L | 4 | 1.00 | 0.00 |
| Occipital_Mid_R | 4 | 1.00 | 0.00 |
| Fusiform_L | 4 | 1.00 | 0.00 |
| Temporal_Pole_Mid_L | 4 | 1.00 | 0.00 |
| Occipital_Inf_L | 3 | 0.33 | 0.67 |
| Occipital_Inf_R | 3 | 0.33 | 0.67 |
| Temporal_Sup_R | 3 | 0.33 | 0.67 |
| Frontal_Mid_L | 3 | 1.00 | 0.00 |
| Frontal_Med_Orb_R | 3 | 1.00 | 0.00 |
| Cingulum_Mid_L | 3 | 1.00 | 0.00 |
| Cingulum_Mid_R | 3 | 1.00 | 0.00 |
| Cingulum_Post_R | 3 | 1.00 | 0.00 |
| Parietal_Inf_R | 3 | 1.00 | 0.00 |
| Paracentral_Lobule_L | 3 | 1.00 | 0.00 |
| Pallidum_L | 3 | 1.00 | 0.00 |
| Pallidum_R | 3 | 1.00 | 0.00 |
| Frontal_Sup_Orb_L | 2 | 1.00 | 0.00 |
| Frontal_Inf_Orb_L | 2 | 1.00 | 0.00 |
| Frontal_Sup_Medial_L | 2 | 1.00 | 0.00 |
| Frontal_Med_Orb_L | 2 | 1.00 | 0.00 |
| Hippocampus_R | 2 | 1.00 | 0.00 |
| Parietal_Sup_L | 2 | 1.00 | 0.00 |
| SupraMarginal_R | 2 | 1.00 | 0.00 |
| Putamen_L | 2 | 1.00 | 0.00 |
| Putamen_R | 2 | 1.00 | 0.00 |
| R: Right, L: Left, Ant: Anterior, Inf: Inferior, Med: Medial, Mid: Middle, Oper: Opercular, Orb: Orbital, Post: Posterior, Sup: Superior, Tri: Triangular part | | | |
